# A Modified Staged Three-Level Pedicle Subtraction Osteotomy Approach for Severe Ankylosing Spondylitis Using CT Navigation and Ultrasonic Bone Scalpel

**DOI:** 10.7759/cureus.101882

**Published:** 2026-01-19

**Authors:** Rawan Masarwa, Nigil S Palliyil, Sultan Al-Kalbani, Hussein Akil, Elie Najjar, Nasir A Quraishi

**Affiliations:** 1 Spine Surgery, Centre for Spinal Studies and Surgery, Queen’s Medical Centre, Nottingham University Hospitals NHS Trust, Nottingham, GBR; 2 College of Medicine, University of Balamand, Balamand, LBN

**Keywords:** bone scalpel pso, multilevel pso, pso in ankylosing spondylitis, sagittal imbalance, spinal deformity correction

## Abstract

Pedicle subtraction osteotomy (PSO) is a well-established technique for correcting rigid spinal deformities but remains technically demanding and carries notable risks. We describe a modified three-level PSO technique utilizing CT-guided navigation and an ultrasonic bone scalpel in a patient with severe sagittal imbalance secondary to ankylosing spondylitis. The procedure achieved satisfactory deformity correction with maintained clinical and radiographic improvement at 24 months. While further experience is needed to assess its broader applicability, this case suggests that incorporating modern navigation and precision tools may help optimize the safety and efficiency of complex PSO procedures.

## Introduction

Restoration of sagittal alignment is a primary consideration when treating adult spinal deformities, as it significantly influences functional outcomes and quality of life [1]. The technique of pedicle subtraction osteotomy (PSO) was first described by Thomasen in 1985 for deformity correction in patients with ankylosing spondylitis (AS) [2]. Since then, PSO has been widely applied to correct fixed sagittal imbalance due to various spinal conditions, including degenerative scoliosis, idiopathic scoliosis, post-traumatic deformities, iatrogenic flatback syndrome, and AS [3]. However, PSO remains technically demanding, with potential complications such as substantial blood loss, neurological deficits, postoperative wound infections, pseudarthrosis, adjacent segment degeneration, and junctional failure [4].

Despite these challenges, PSO offers a powerful correction for sagittal (and coronal) deformities without requiring additional anterior approaches. Typically, PSO can achieve between 25° and 40° of lordosis at each osteotomy level, depending on the wedge resection dimensions, and aims for solid bone-on-bone contact across all three spinal columns [4].

However, detailed descriptions of planning, level selection, and execution strategies for more extensive staged corrections remain limited. The present report describes a staged three-level PSO in a single patient, focusing on technical considerations and decision-making.

In this article, we describe a modified PSO technique using CT navigation and an ultrasonic bone scalpel (BoneScalpel; Misonix, Farmingdale, NY, USA), performed at three levels to correct severe sagittal imbalance in a patient with AS. This technique is illustrated with sawbone models and clinical images from a case with fixed sagittal deformity and “chin-on-chest” deformity, highlighting the precision and effectiveness of this approach.

## Case presentation

**Figure 1:**
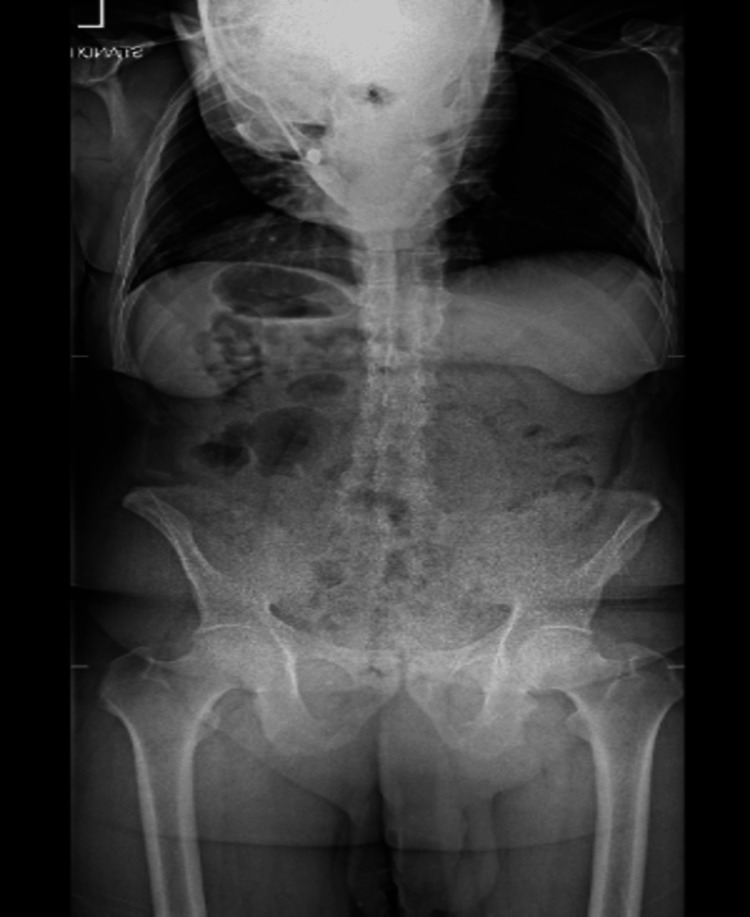
Anteroposterior upright radiograph of the whole spine

**Figure 2:**
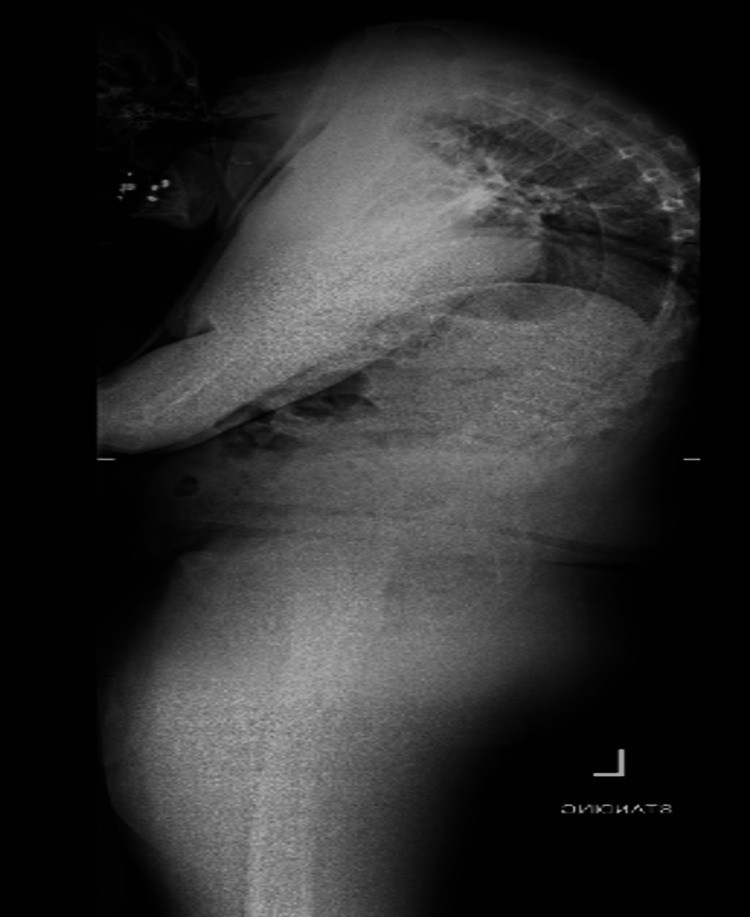
Upright radiograph of the whole spine, lateral view, demonstrating a rigid cervicothoracic deformity

**Figure 3:**
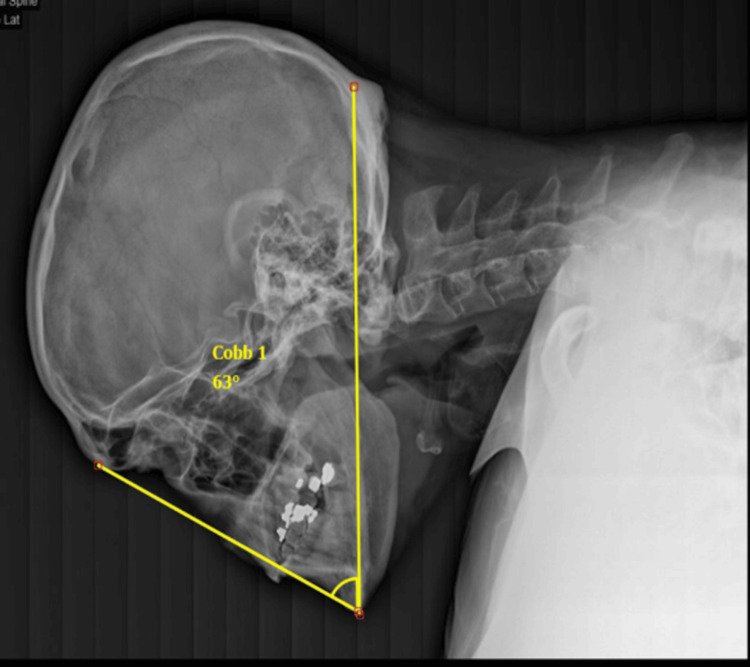
Lateral radiograph of the skull and cervical spine demonstrating a CBVA of 63 degrees CBVA: chin-brow vertical angle

This case involves a 38-year-old male with severe AS complicated by morbid obesity (BMI = 44 kg/m^2^), who presented with a debilitating "chin-on-chest" deformity. This deformity severely affected his functional abilities, limiting his forward gaze, mobility, and daily activities. Upright radiographs showed a rigid cervico-thoracic kyphotic deformity with positive sagittal balance (Figures 1-2). His chin-brow vertical angle (CBVA) was measured at 63°, indicating substantial forward tilt and impaired visual alignment (Figure 3).

**Figure 4:**
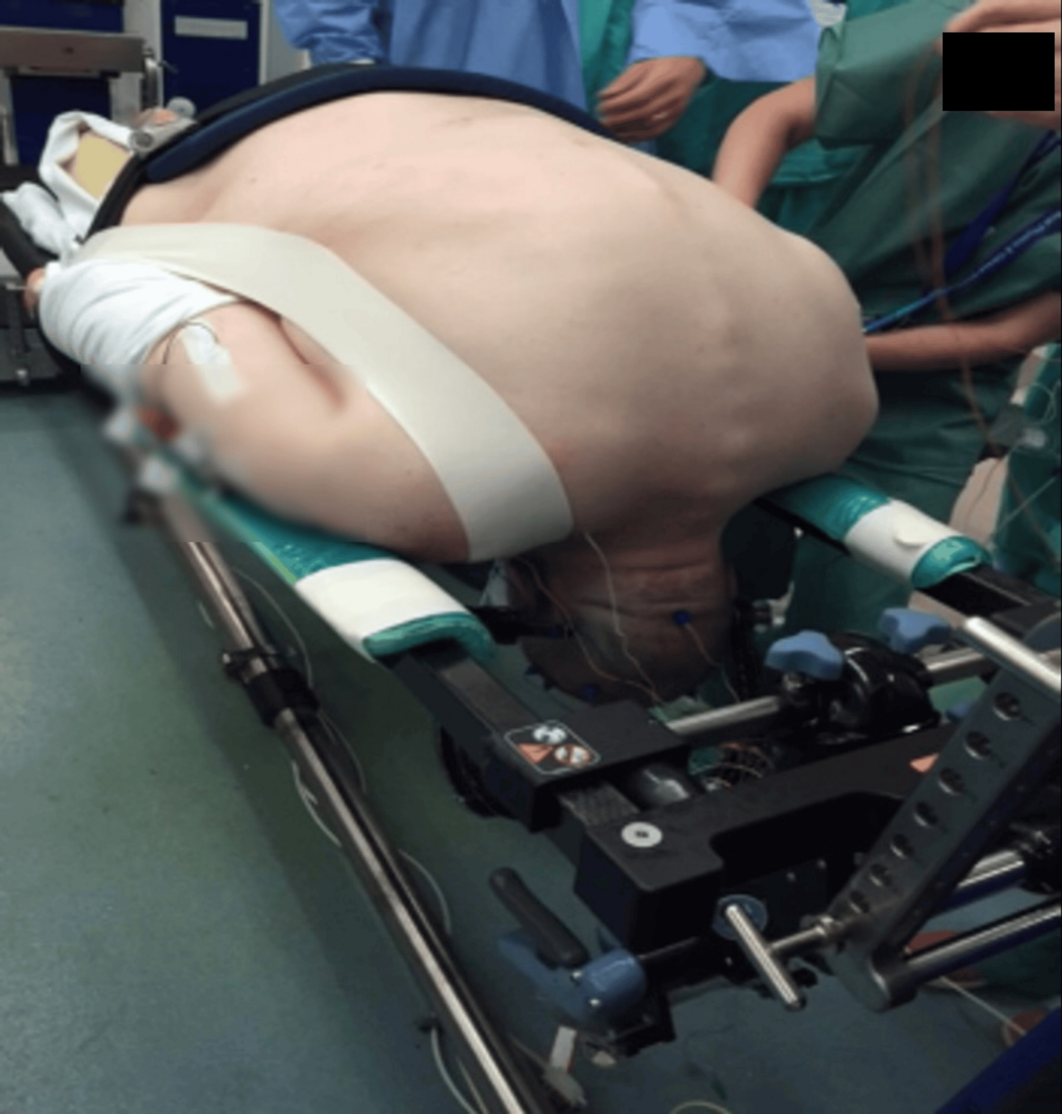
The patient underwent fiber-optic intubation and was then carefully positioned in the reverse Trendelenburg position, with the head supported using a Mayfield clamp and adequate padding of pressure points

**Figure 5:**
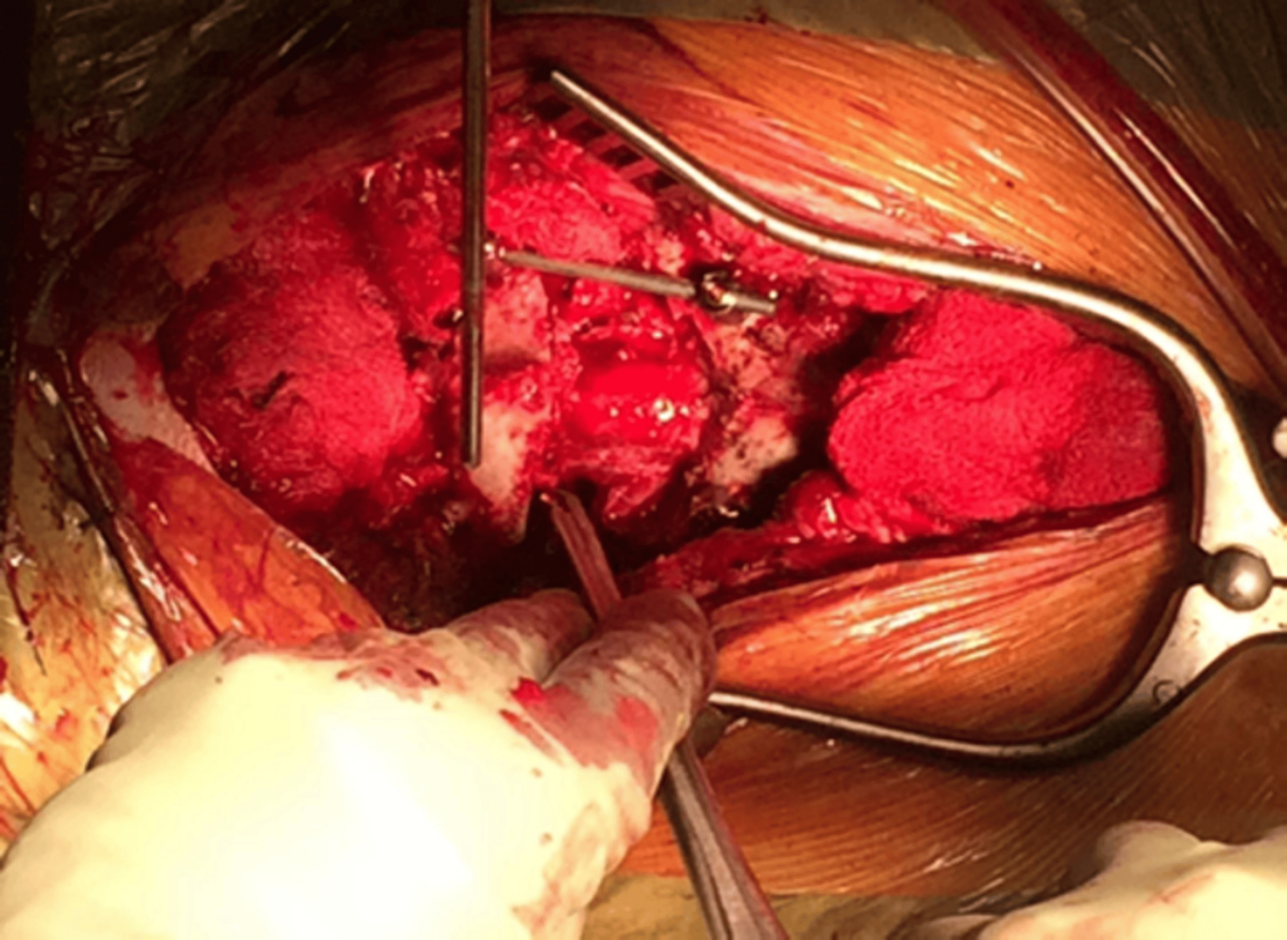
Decancellation of the T1 vertebral body performed through the window created after resection of the pedicles

**Figure 6:**
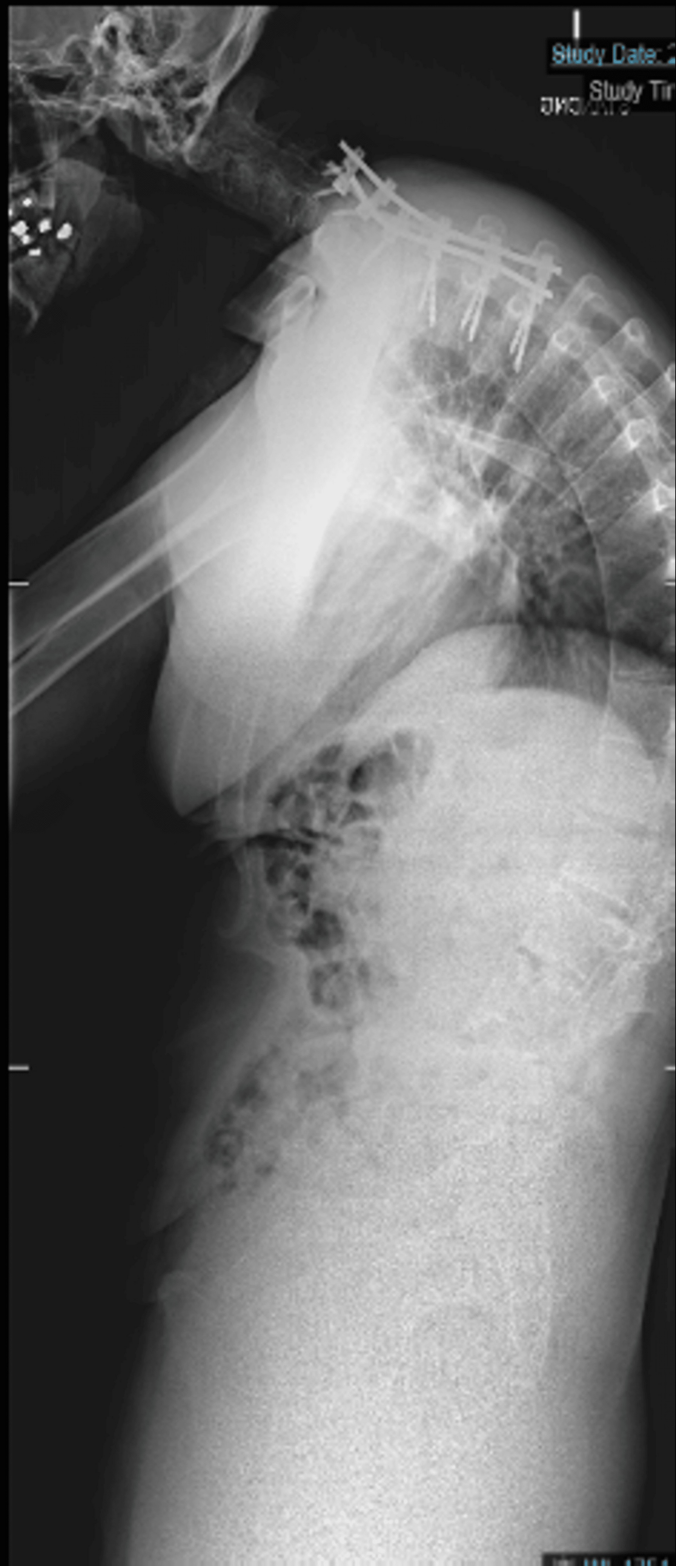
Lateral upright radiograph demonstrating improvement in sagittal alignment following T1 PSO PSO: pedicle subtraction osteotomy

**Figure 7:**
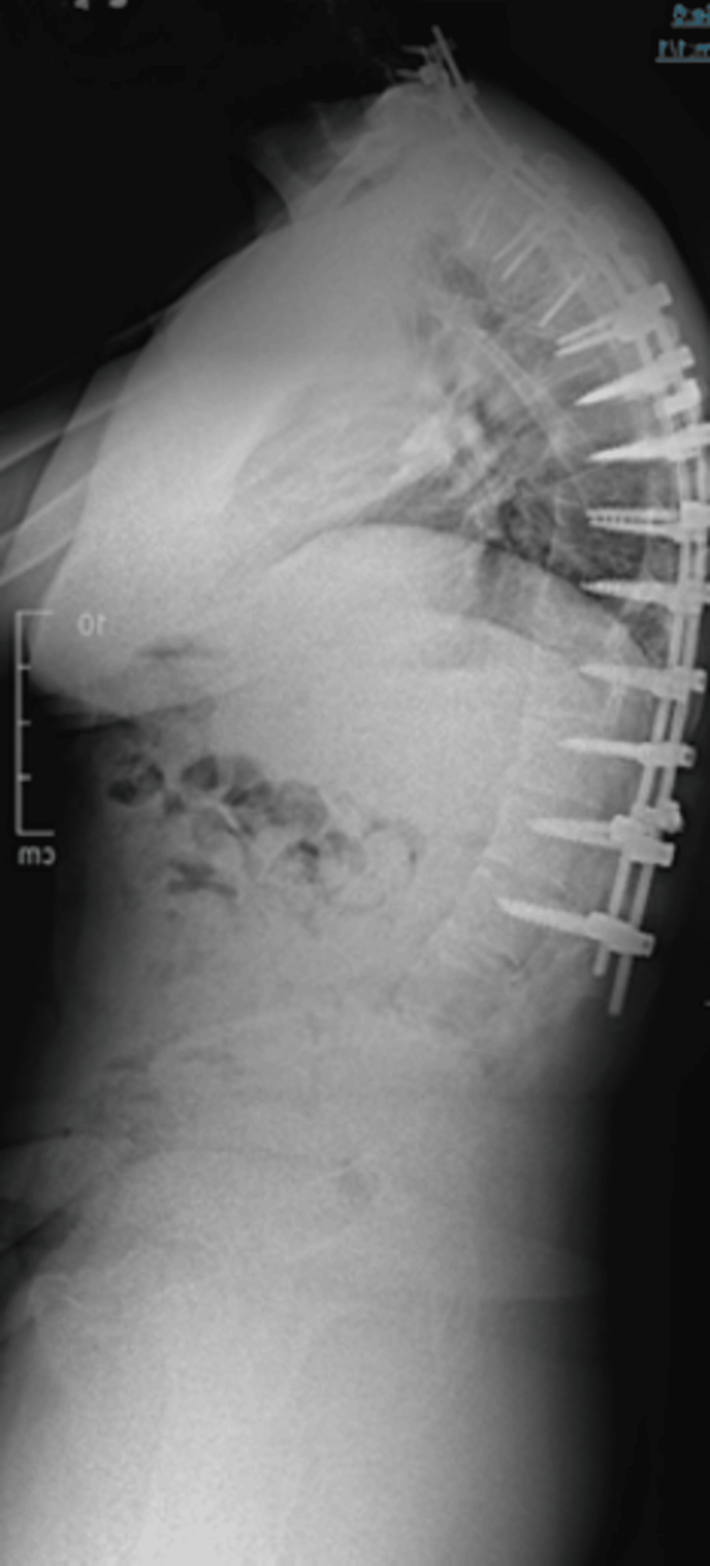
Lateral upright radiograph demonstrating improvement in sagittal alignment following T10 PSO PSO: pedicle subtraction osteotomy

The patient underwent a staged, three-level PSO at T1, T10, and L4, with posterior instrumented fusion extending from C5 to the pelvis, supported by O-arm imaging and intraoperative neuromonitoring. The first stage (T1 PSO with C5-T4 fusion) focused on correcting the upper deformity at T1 and stabilizing the C5-T4 region. The patient was intubated using fibre optic techniques and carefully positioned (Figures 4-6). Screws were placed from C4 to T4, followed by a T1 laminectomy and C7/T2 laminotomies. The first rib head was osteotomized, and the C8 and T1 nerve roots were isolated and protected. Both T1 pedicles were skeletonized, and the ultrasonic bone scalpel was used to perform osteotomies into the vertebral bodies. Temporary rods were placed, and decancellation of the T1 vertebral body was completed (Figure 7), followed by perforation of the posterior vertebral cortex.

Osteotomy closure was performed in a gradual and guarded manner, with controlled extension assisted via the Mayfield clamp (Integra LifeSciences, Princeton, NJ, USA) under continuous intraoperative neuromonitoring. This maneuver was carefully titrated and used to fine-tune alignment rather than to apply uncontrolled corrective force. Final contoured rods were placed, completing the T1 correction.

At the thoracic level (T10), the osteotomy was performed using the same principles of controlled decancellation and guarded closure under continuous neuromonitoring. In addition, a bilateral costotransversectomy with rib head resection was performed to obtain safe lateral access to the vertebral body and facilitate controlled wedge resection while protecting the pleura. Wider posterior decompression was undertaken to allow safe accommodation of the thoracic spinal cord during osteotomy closure. Closure was then completed in a stepwise manner using temporary rod stabilization and gradual compression, as described above.

**Figure 8:**
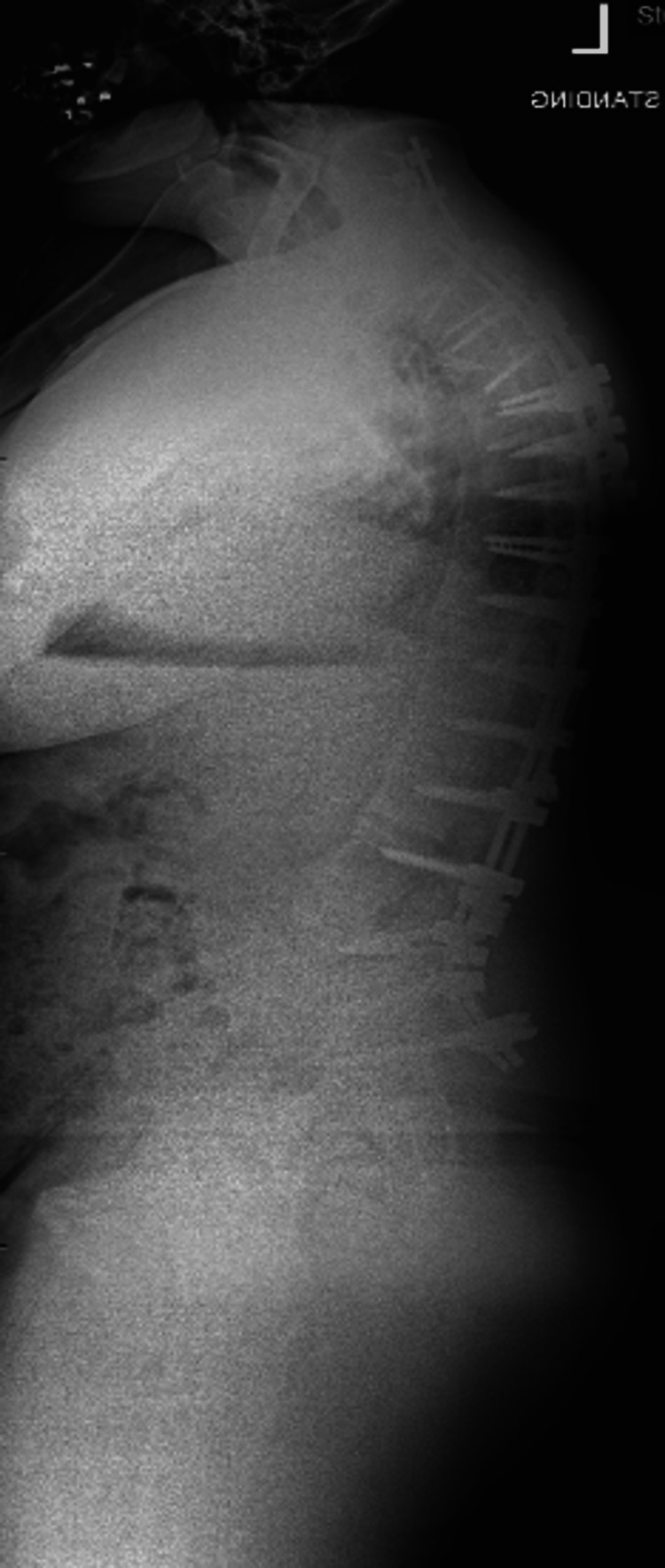
Lateral upright radiograph demonstrating improvement in sagittal alignment following L4 PSO PSO: pedicle subtraction osteotomy

**Figure 9:**
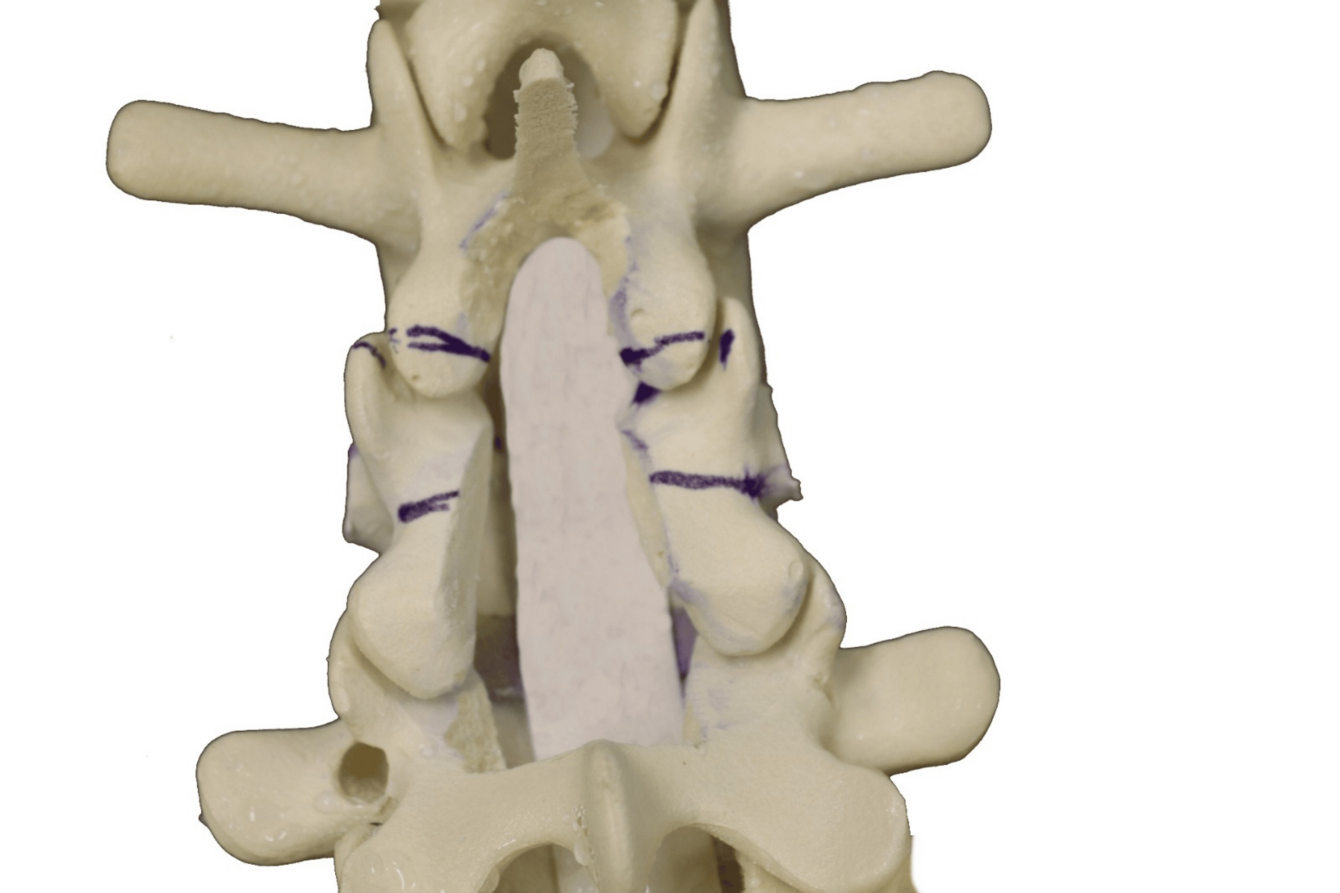
Marking of bone cuts using an ultrasonic bone scalpel, followed by central laminectomy and laminotomies, with markings for planned facet cuts

**Figure 10:**
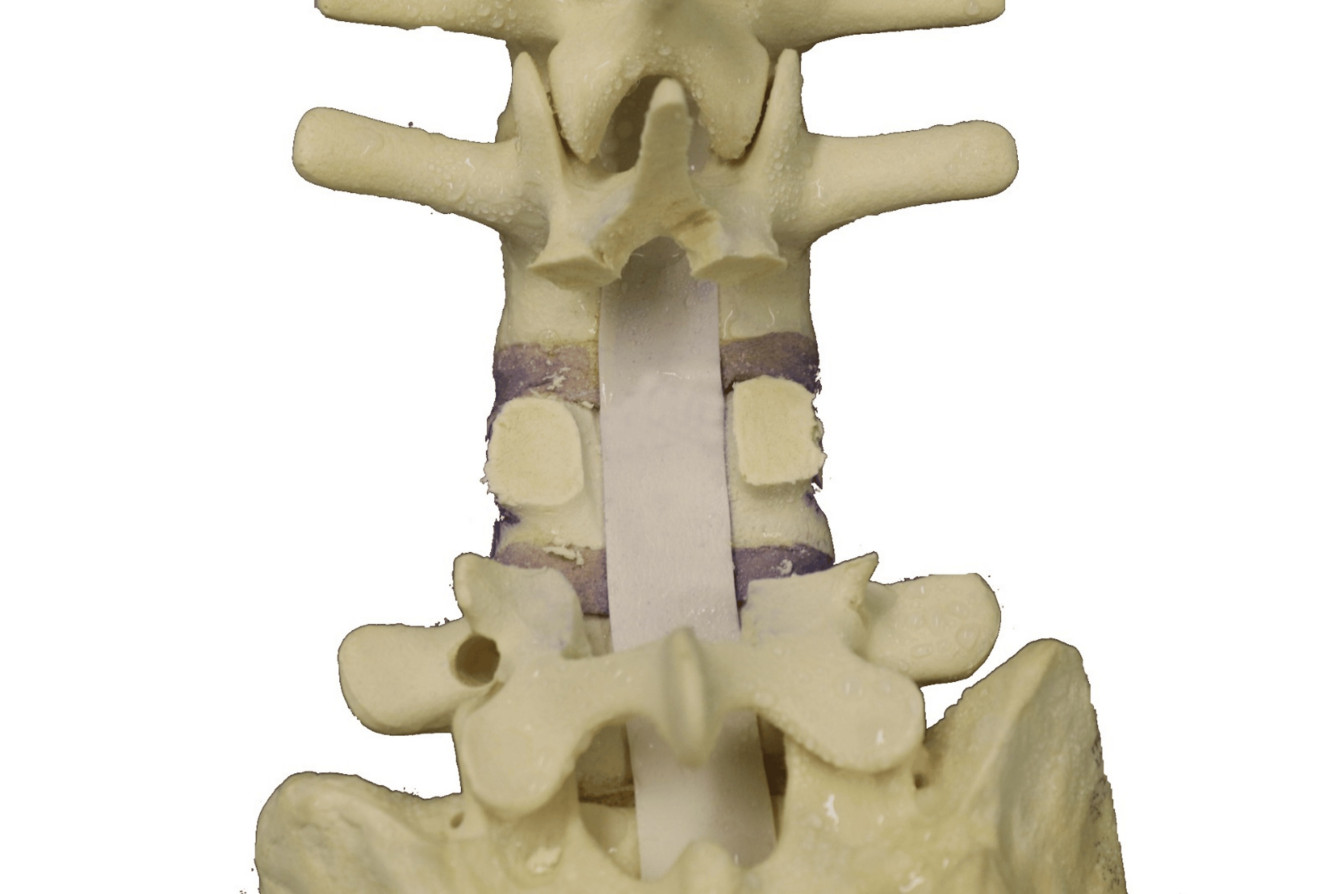
The pedicles are effectively “skeletonized”

Each subsequent stage, at T10 and L4, was spaced approximately three months apart to allow for recovery and stability assessment. Instrumentation was extended to the pelvis, and successive corrections are shown in Figures 8-10.

The staged PSO approach successfully restored sagittal alignment, with marked improvement in the patient’s forward gaze and functional posture. Objective radiographic analysis demonstrated progressive correction of sagittal alignment and horizontal gaze across the staged procedure (Table 1). The CBVA improved from 63° pre-operatively to 5° at final follow-up. Sagittal vertical axis improved from 117 mm pre-operatively to 6 mm following completion of all three osteotomies. Thoracic kyphosis was reduced from 70° to 34°, while lumbar lordosis improved from 19.7° to 32.9° after the L4 PSO. Operative duration ranged from 4.0 to 5.5 hours per stage, with estimated blood loss between 500 and 750 mL (Table 2). The Oswestry Disability Index (ODI) improved significantly, decreasing from 70% preoperatively to 38% at 24 months postoperatively. The patient experienced minor residual numbness along the C8 distribution in his left hand, though the remainder of his neurological examination was unremarkable.

**Table 1:** Sequential spinopelvic and gaze parameters across staged correction Sequential radiographic correction of sagittal alignment and horizontal gaze following staged pedicle subtraction osteotomies (PSOs) at T1, T10, and L4. Measurements are shown pre-operatively, after each corrective stage, and at final follow-up.

Stage	Lumbar Lordosis (LL) in °	Thoracic Kyphosis (TK) in °	Sagittal Vertical Axis (SVA) in mm	Chin-Brow Vertical Angle (CBVA) in °
Pre-operative	19.7	70.0	117	63
After T1 PSO	19.7	60.7	48	38
After T10 PSO	16.0	34.0	9.2	7
Final (After L4 PSO)	32.9	34.0	6	5

**Table 2:** Operative duration and estimated blood loss by surgical stage Operative duration and estimated blood loss for each staged pedicle subtraction osteotomy (PSO). Values are representative of intraoperative anesthetic records. Staging was employed to mitigate cumulative physiological stress and allow interval recovery between procedures.

Surgical Stage	Operative Time (Hours)	Estimated Blood Loss (mL)
T1 PSO (C5-T4 Fusion)	5.5	750
T10 PSO	4.5	600
L4 PSO (Extension to Pelvis)	4.0	500

The operative stages followed a modified PSO technique, which is described in detail below to illustrate the procedural sequence and key technical refinements.

The PSO procedure began with exposure of the L4 vertebra’s transverse processes using monopolar diathermy. These processes were carefully dissected free and osteotomized at their base with the ultrasonic bone scalpel. The lateral aspect of the vertebral body was then exposed through blunt dissection using a curved Cobb instrument and swabs, with each lateral gutter packed with a swab for protection and hemostasis.

**Figure 11:**
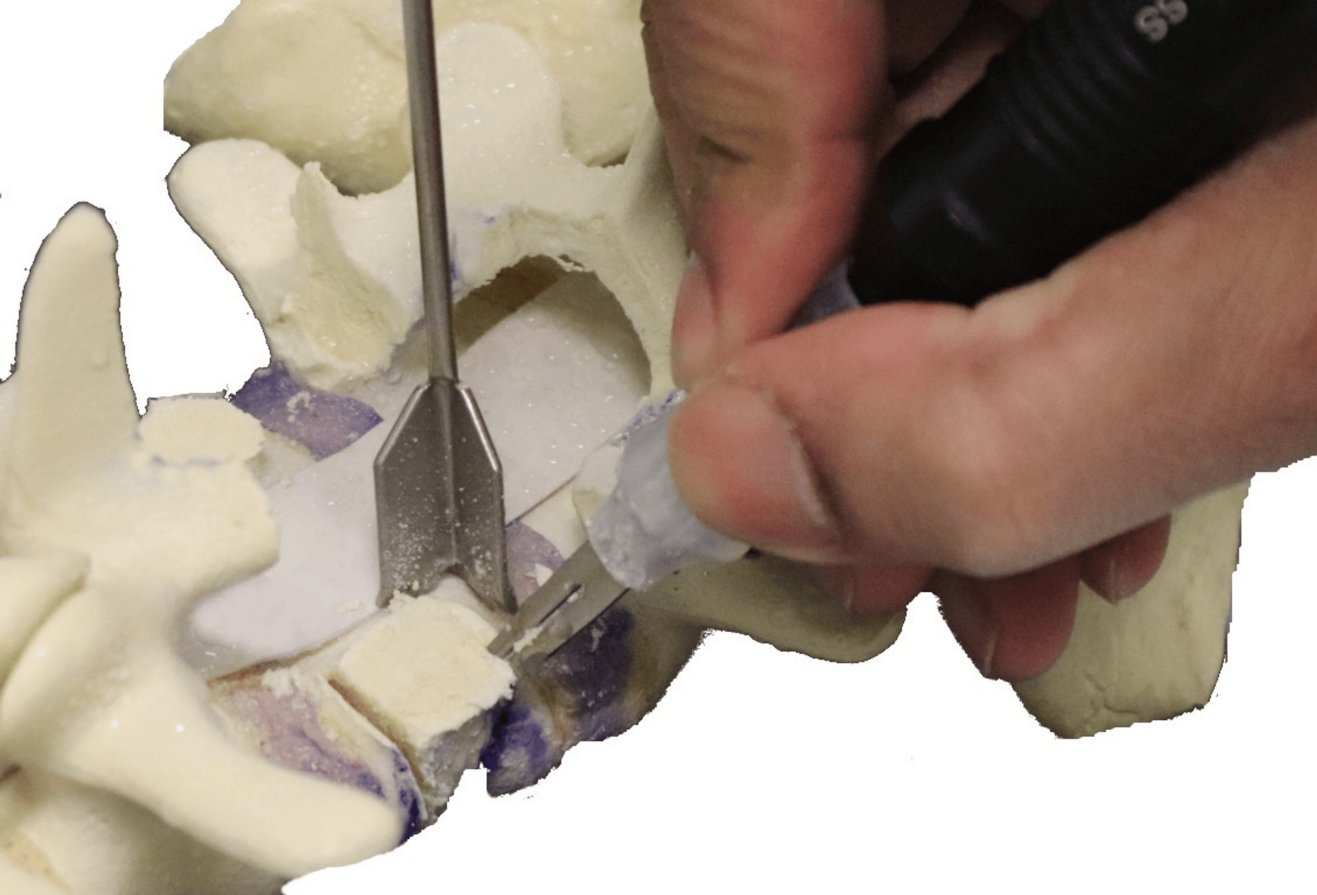
Superior bone cut performed just above the pedicle

At the target level (L4), a partial laminectomy was performed, removing the inferior half of the superior lamina and the superior half of the inferior lamina (Figure 11). The BoneScalpel was used to make precise cuts, removing the central slips en bloc when possible. The ligamentum flavum was carefully released from the lamina to preserve the underlying dura. Navigation guidance was relied upon during these steps, particularly in cases where anatomical landmarks were obscured due to AS.

**Figure 12:**
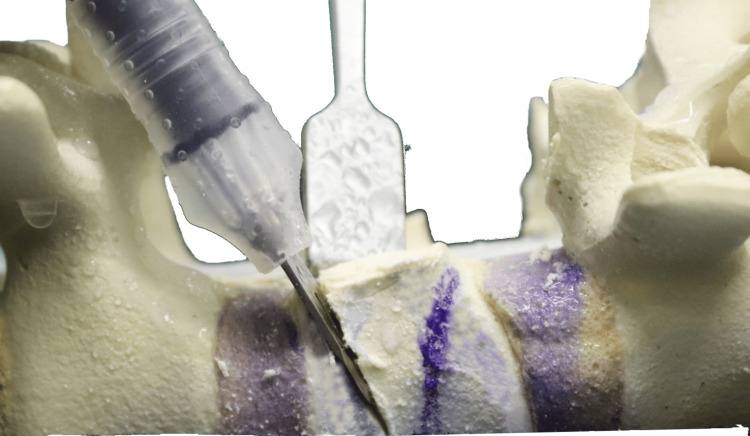
Inferior bone cut performed just below the pedicle

Following laminectomy, the pedicles were skeletonized (Figure 12). Work began on the left side, starting with a cut below the pedicle to decompress the exiting nerve root, followed by a cut above the pedicle to release the traversing nerve root. The pedicle bulk was reduced using rongeurs down to the pedicle base, and the medial wall of the pedicle was removed to complete the skeletonization. The area was packed with a swab and hemostatic agent before repeating the same process on the right side.

**Figure 13:**
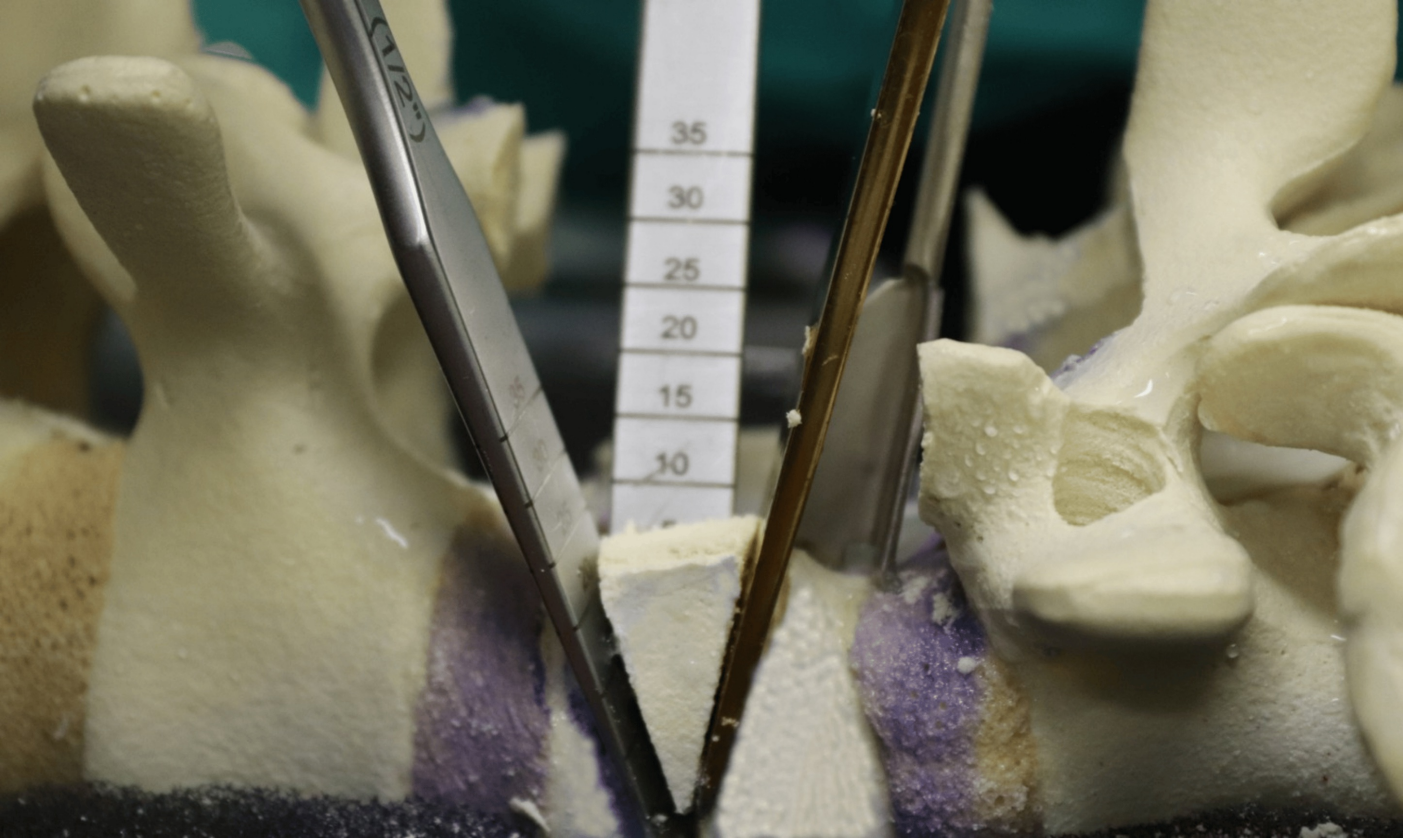
Osteotomes used to “deliver” the wedged bone segment

**Figure 14:**
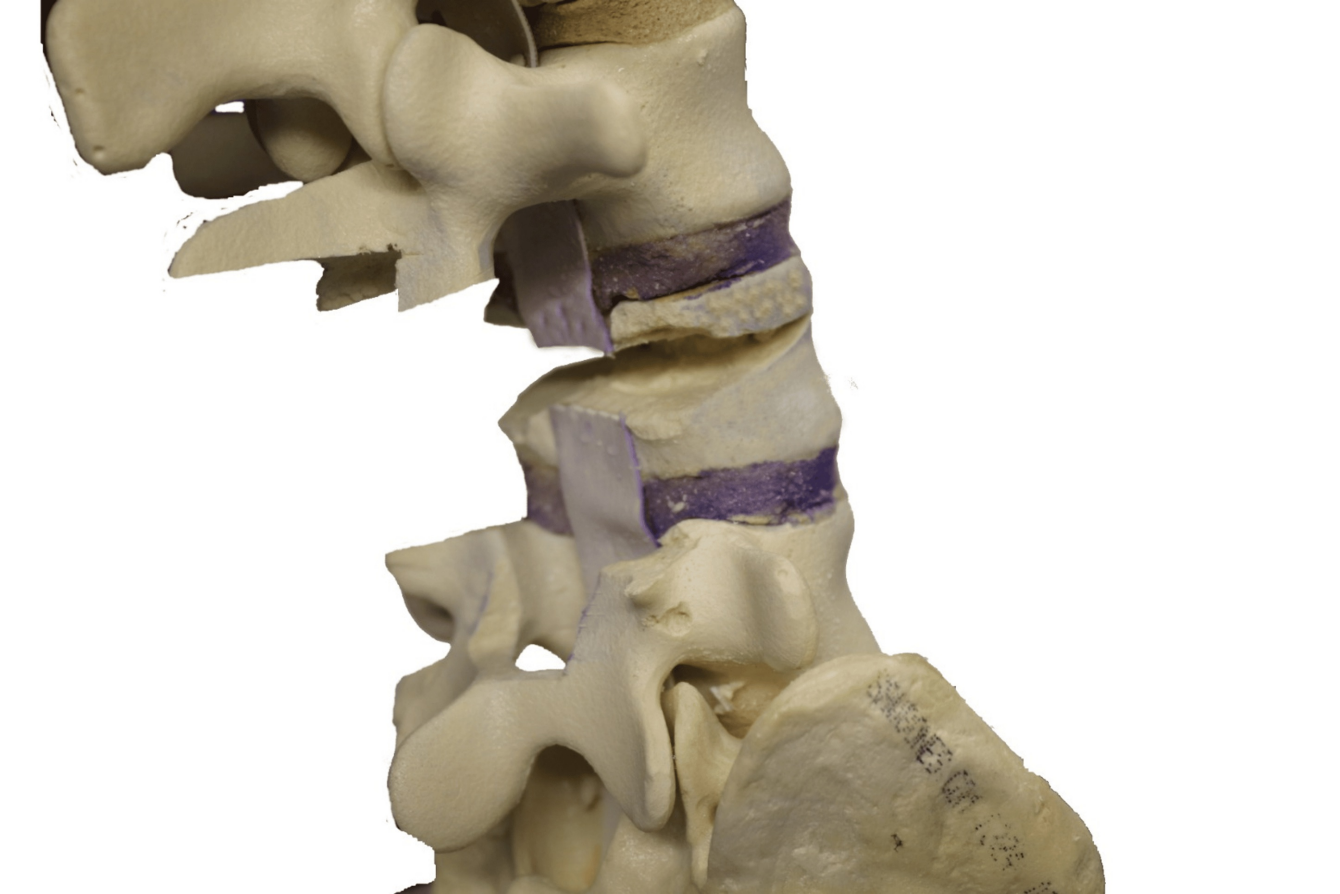
Final result showing the extent of the pedicle cuts and decancellation

Pedicle cuts were then made on the left side using the ultrasonic bone scalpel (Figures 13-14), with a nerve root retractor placed to protect the dura during these cuts. Curved spoon retractors were inserted subperiosteally on both sides of the vertebral bodies to expose the area for further work. The lateral cortices of the vertebral bodies were removed while preserving the anterior cortex, and navigation was used to verify the adequacy of the cuts.

**Figure 15:**
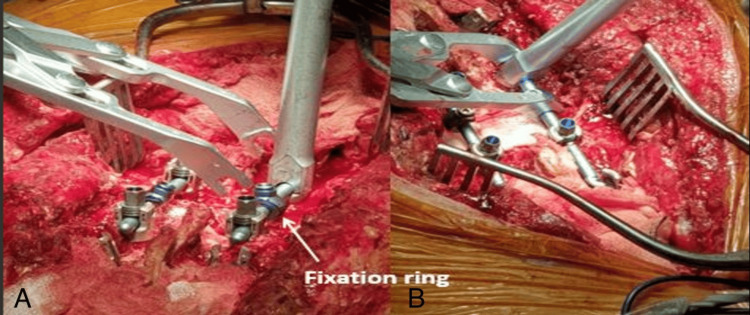
(A) Fixation ring (arrow); (B) compressor positioned to close the osteotomy

With the spoon retractors in place, the wedge segment was released using osteotomes and subsequently removed (Figure 15). Curettes were used to decancellate the vertebral body along the bone scalpel cuts, and the area was packed with a swab before inserting a temporary rod. The same steps were then carried out on the opposite side.

Further removal of cancellous bone was performed to thin the posterior cortex. A transforaminal lumbar interbody fusion (TLIF) rasper was inserted to connect both sides, followed by placement of a second temporary rod. A hockey punch was then used beneath the anterior dura and posterior vertebral cortex to gently fracture the posterior cortex into the vertebral body, and any resulting fragments were removed.

Osteotomy closure was initiated once adequate decompression of the thecal sac and nerve roots had been confirmed. Gradual, controlled closure was achieved using a compressor and fixation ring from the USS II system (DePuy Synthes, Oberdorf, Switzerland), with temporary rod stabilization maintaining alignment throughout the process. No breakable table mechanism was used. Instead, controlled closure relied on sequential segmental compression across the osteotomy site combined with appropriate patient positioning and spacing of chest and pelvic bolsters, allowing the osteotomy to close in a controlled manner under gravity.

Intraoperative motor-evoked potentials (MEPs) were rechecked at regular intervals, approximately every few millimeters of closure. Upon completion, the dural sac and nerve roots were reassessed to ensure no compression, and an intraoperative lateral radiograph in the prone position was obtained to confirm adequacy of osteotomy closure and local sagittal contour at the index level. Additional bone resection was performed if further closure was required. Definitive sagittal balance assessment was performed on postoperative standing full-length radiographs.

Temporary rods were then replaced with permanent rods, or permanent rods were added alongside them. A three- or four-rod construct could be used depending on surgeon preference, although this was not considered essential in AS cases due to the rapid fusion typically observed.

Finally, the wound was thoroughly debrided with povidone iodine and saline. The posterior elements were decorticated, and a bone graft consisting of autograft and demineralized bone matrix was overlaid on the lamina, facets, and transverse processes. A deep drain was inserted, and the wound was closed in layers with secure fascial closure. The patient was mobilized from the first postoperative day as tolerated and was provided with a thoraco-lumbo-sacral orthosis for three months postoperatively.

## Discussion

This modified PSO technique, combining the precision of an ultrasonic bone scalpel with CT-guided navigation, has proven effective in addressing complex sagittal deformities and was associated with acceptable operative duration and blood loss in this individual case. The senior author has successfully applied this approach in over 50 PSO procedures, underscoring its reliability and clinical benefits.

The concept of osteotomy for spinal deformity correction has evolved significantly. Initially introduced by Smith-Petersen et al. in 1945 [5] and later refined by Thomasen in 1985 for AS, the PSO achieved segmental corrections of 12°-50° with minimal severe complications in Thomasen’s series of Bechterew’s syndrome cases [2].

Preoperative planning in this case was based on clinical assessment and full-length standing radiographs rather than formal deformity-planning software. The correction strategy was guided by global sagittal balance, horizontal gaze requirements, deformity apex, and regional rigidity. The decision to include a thoracic PSO at T10 reflected the presence of a rigid thoracic kyphotic component contributing to persistent sagittal imbalance after cervicothoracic correction, and allowed distribution of angular correction across spinal regions rather than overloading a single level.

The ultrasonic bone scalpel from BoneScalpel provides several distinct advantages that make it highly valuable in spinal and neurosurgical procedures. Operating at an oscillation frequency of 22.5 kHz, it allows for precise, targeted cuts that selectively affect mineralized tissue while minimizing bone debris and the risk of mechanical injury to adjacent structures. Its atraumatic effect on soft tissue is particularly beneficial in narrow epidural spaces, where conventional tools may increase the likelihood of durotomy or nerve injury. Furthermore, by reducing bleeding at the osteotomy sites, the bone scalpel helps maintain a clear surgical field, thereby improving intraoperative visualization and overall surgical safety [6,7].

Intraoperative navigation provides essential three-dimensional feedback, particularly in severe kyphosis, where landmarks are obscured. The navigation system allows precise orientation of cuts and prevents unintended cortical breaches [8].

Morbid obesity presented additional technical considerations in this case. In patients with a high BMI, intraoperative CT-based navigation can be affected by reduced signal-to-noise ratio and image scatter, potentially limiting accuracy. To mitigate this, meticulous patient positioning, careful reference array placement, and repeated verification of navigational accuracy against exposed anatomical landmarks were employed at each stage. When feasible, short periods of controlled ventilatory pause during image acquisition were used to reduce motion artifact.

Prone positioning in the context of severe AS and obesity also requires particular attention to airway security and physiological optimization. Fibre-optic intubation was used, and positioning was performed with generous chest and pelvic bolstering to minimize abdominal compression, reduce venous congestion, and limit intra-abdominal pressure. Surgeons attempting to replicate this approach should anticipate these challenges and plan positioning, imaging, and anesthetic strategies accordingly.

Despite the potential for transformative outcomes, spinal deformity corrections in AS remain high-risk procedures. Complication rates are substantial, with major complications reported in up to one-third of thoracolumbar cases and 60% in cervical deformities, where neurological complications can reach 25% [9-13]. Known risk factors include advanced age, multilevel PSOs, and extensive corrections [14]. The psychological and physical toll on patients underscores the value of a procedure that can deliver reliable outcomes with minimized risks.

This report has several limitations inherent to a single-case design. In particular, while the ODI was available and demonstrated meaningful functional improvement, additional multidimensional patient-reported outcome measures (PROMs) such as the Visual Analog Scale (VAS), EuroQol-5 Dimensions (EQ-5D), Short Form-36 (SF-36), and Neck Disability Index were not collected in a standardized fashion. Future studies and larger series would benefit from a comprehensive PROM assessment to better capture pain, health-related quality of life, and cervical-specific functional outcomes following complex deformity correction.

While two-level osteotomies have been reported in AS, to our knowledge, this represents a description of a staged three-level PSO in a single patient. This case illustrates the technical feasibility of such an approach in carefully selected patients with severe, rigid deformity, while acknowledging that further experience is required before broader conclusions can be drawn.

## Conclusions

Complication prevention remains a central concern in complex spinal deformity surgery, particularly in patients with AS. In this case, the use of O-arm navigation provided precise, real-time three-dimensional visualization that helped address the distorted anatomy often encountered in such patients. The ultrasonic bone scalpel facilitated controlled bone removal and reduced mechanical manipulation near neural structures. While these technologies appeared to support procedural accuracy and safety in this individual case, further experience and larger series are needed to determine their broader applicability and impact on surgical outcomes.
